# Gametocidal Factor Transferred from *Aegilops geniculata* Roth Can Be Adapted for Large-Scale Chromosome Manipulations in Cereals

**DOI:** 10.3389/fpls.2017.00409

**Published:** 2017-03-27

**Authors:** Michał T. Kwiatek, Halina Wiśniewska, Aurelia Ślusarkiewicz-Jarzina, Joanna Majka, Maciej Majka, Jolanta Belter, Hanna Pudelska

**Affiliations:** ^1^Cereal Genomics Team, Department of Genomics, Institute of Plant Genetics, Polish Academy of SciencesPoznań, Poland; ^2^Bioengineering Team, Department of Biotechnology, Institute of Plant Genetics, Polish Academy of SciencesPoznań, Poland; ^3^Cytogenetics and Molecular Physiology of Plants Team, Department of Environmental Stress Biology, Institute of Plant Genetics, Polish Academy of SciencesPoznań, Poland

**Keywords:** *Aegilops geniculata*, androgenesis, chromosome aberrations, doubled haploids, gametocidal factor, meiosis, segregation distortion, triticale

## Abstract

Segregation distorters are curious, evolutionarily selfish genetic elements, which distort Mendelian segregation in their favor at the expense of others. Those agents include gametocidal factors (Gc), which ensure their preferential transmission by triggering damages in cells lacking them via chromosome break induction. Hence, we hypothesized that the gametocidal system can be adapted for chromosome manipulations between *Triticum* and *Secale* chromosomes in hexaploid triticale (×*Triticosecale* Wittmack). In this work we studied the little-known gametocidal action of a Gc factor located on *Aegilops geniculata* Roth chromosome 4M^g^. Our results indicate that the initiation of the gametocidal action takes place at anaphase II of meiosis of pollen mother cells. Hence, we induced androgenesis at postmeiotic pollen divisions (via anther cultures) in monosomic 4M^g^ addition plants of hexaploid triticale (AABBRR) followed by production of doubled haploids, to maintain the chromosome aberrations caused by the gametocidal action. This approach enabled us to obtain a large number of plants with two copies of particular chromosome translocations, which were identified by the use of cytomolecular methods. We obtained 41 doubled haploid triticale lines and 17 of them carried chromosome aberrations that included plants with the following chromosome sets: 40T+Dt2RS+Dt2RL (5 lines), 40T+N2R (1), 38T+D4RS.4BL (3), 38T+D5BS-5BL.5RL (5), and 38T+D7RS.3AL (3). The results show that the application of the Gc mechanism in combination with production of doubled haploid lines provides a sufficiently large population of homozygous doubled haploid individuals with two identical copies of translocation chromosomes. In our opinion, this approach will be a valuable tool for the production of novel plant material, which could be used for gene tracking studies, genetic mapping, and finally to enhance the diversity of cereals.

## Introduction

Chromosomal rearrangements play a key role in the evolution of living organisms. Such changes affect the biology of organisms and can result in development of new phenotypes or can even lead to speciation (Levin, 2004). Biological processes, such as mechanisms of variation and selection, are widely used in science for this purpose. In this vein, chromosomal rearrangements are induced to improve the genetic variability of crops, including cereals. An acceleration of the breeding process can be achieved using chromosome manipulations. This could be induced in several ways, i.e., modification of the chromosome pairing mechanism (*Ph* locus) (Riley and Chapman, 1958; Griffiths et al., 2006), irradiation (Sears, 1981), somaclonal variation (Larkin and Scowcroft, 1981), or use of gametocidal factors (Endo et al., 1988; Endo, 2007, 2015), followed by the respective crossing program.

Gametocidal (Gc) factors belong to the group of segregation distorters, which are involved in speciation by triggering reproductive isolation. Segregation distortion is described as a phenomenon where one of the alleles at a heterozygous locus is transmitted at a higher frequency than the expected Mendelian ratio (Sandler and Novitski, 1957; Sandler et al., 1959). Segregation distorters have been found in many animals, plants and fungi (Burt and Trivers, 1998). Many different types of segregation distorters have been reported in plants, e.g., a pollen killer in *Nicotiana* (Cameron and Moav, 1957), gamete eliminator in *Solanum lycopersicum* (Rick, 1966), *Ki* allele in *Triticum* (Nyquist, 1962), gametocidal factor in *Aegilops* (Endo and Tsunewaki, 1975), egg killer in *Oryza* (Ikehashi and Araki, 1986), *Sd-1* segregation distorter in *Agropyron* (Dvořák and Appels, 1986), B-chromosomes in many animal, fungi, and plant species (Jones, 1991), chromosomal knobs in *Zea mays* (Kanizay et al., 2013), and female meiotic drive in *Mimulus* (Finseth et al., 2015).

Gc factors are selfish genetic elements that ensure their preferential transmission by inducing chromosome breaks in gametophytes lacking them (Nasuda et al., 1998). Some Gc factors have been reported and assigned to particular chromosomes within the *Aegilops* genus: *Aegilops caudata* L. and *Ae. triuncialis* L. (chromosome 3C), *Ae. cylindrica* Host (2C), *Ae. speltoides* Tausch (2S and 6S), *Ae. longissima* Schweinf. & Muschl. and *Ae. sharonensis* Eig. (2S and 4S), and *Ae. geniculata* Roth (4M) (Endo, 2007). Gc-carrying chromosomes have been incorporated from *Aegilops* species into bread wheat (*Triticum aestivum* L.) during the production of chromosome addition lines and alloplasmic lines (Endo, 1990). Certain *Aegilops* chromosomes were not removed during backcrossing and led to a conclusion that there is a gametocidal action of sporophytes carrying *Aegilops* chromosomes, which consist in inducing chromosome breaks in gametes lacking the Gc chromosome (Endo and Tsunewaki, 1975; Maan, 1975). The gametes are non-functional if the breakage is severe, but in certain situations the chromosome aberrations—including deletions, translocations, ring, and telocentric chromosomes—are not sufficient to kill the gamete, hence it may still function and be transmitted to the offspring (Tsujimoto and Tsunewaki, 1985). This phenomenon was applied to produce deletion stocks of common wheat (Endo and Gill, 1996). Moreover, a Gc system has been established in wheat-barley addition lines to produce barley dissection lines of wheat, bearing dissected barley chromosomes (Shi and Endo, 1997, 1999, 2000). This approach made it possible to construct cytological chromosome maps or to localize genes, DNA markers or chromosome breakpoints (Nasuda et al., 2005b; Sakata et al., 2010; Ishihara et al., 2014).

Induction of new genetic variability is crucial for cereal breeding, especially for bread wheat (*Triticum aestivum* L., 2*n* = 6*x* = 42, AABBDD). Species that are close relatives of wheat, such as rye (*Secale cereale* L., 2*n* = 2*x* = 14, RR), offer vast germplasm pools for various agronomic and quality traits, such as resistance or tolerance to pests, diseases, and adverse environmental conditions. Wheat-rye hybrids are widely used to study genetic mechanisms of wheat, such as homeologous chromosome pairing (Lukaszewski and Kopecky, 2010; Martín et al., 2014). Furthermore, wheat/rye chromosome translocations are interesting, considering their association with novel phenotypes or their usefulness in genetic mapping (Bhat et al., 2007). Those efforts succeeded by achieving wheat-rye chromosomal translocations and substitutions, which were commercialized with a great success. Moreover, a fusion of wheat and rye genomes allowed breeders to obtain an artificial cereal called triticale (×*Triticosecale* Wittmack), which after many efforts is competing with wheat as a commercial cereal. Triticale is an important source of germplasm for wheat improvement, providing a platform to transfer valuable rye traits to wheat.

One of the crucial issues for crop improvement is an acceleration of breeding progress. Doubled haploid (DH) breeding is the most productive way to incorporate new genes or loci for crop improvement. Plant breeders seek to bypass sexual reproduction, to produce DH individuals derived from the chromosome-doubled cells of the haploid gametophyte (Kelliher et al., 2017). The key role of DH production is a fixation of recombinant haploid genomes in inbred lines (Chang and Coe, 2009). The big advantage of DHs in breeding lines is the shorter time needed between the initial cross and large-scale testing of developed lines. This approach allows us to provide large amounts of seed material for replicated field trials. What is more, the crucial attribute of DHs is that they are perfectly homozygous lines. DH lines can be produced in several ways, applying distant hybridization, androgenesis, or gynogenesis (Forster et al., 2007). In triticale, generally, androgenesis is induced, using anther cultures (Ślusarkiewicz-Jarzina and Ponitka, 2003; Oleszczuk et al., 2011). This technique, in most cases, produces reasonable yields of haploids and DHs and is routinely used in triticale breeding.

Here we investigate the gametocidal action of the 4M^g^ chromosome (derived from *Aegilops geniculata* Roth) during the meiosis of pollen mother cells (PMCs) of monosomic 4M^g^-addition triticale (M4M^g^A) plants. Furthermore, we study the variability of chromosome aberrations that appeared between the A- or B-genome (*Triticum*) and the R-genome (*Secale*) chromosomes, induced by the gametocidal factor and maintained by the production of DH triticale lines.

## Methods

### Plant material and culture conditions

Seeds of monosomic addition hexaploid triticale plants, carrying a 4M^g^ chromosome from *Aegilops geniculata* (Kwiatek et al., 2016c), were germinated on wet filter paper in the dark at room temperature for 4 days. Thereafter the plantlets were transferred to soil and cultivated for 6–8 weeks under short-day conditions (8 h light/16 h dark, 20°/18°C). Finally, the plants were transferred for 6 weeks to vernalizing conditions (10 h light/14 h dark, 4°C) and then returned to long-day conditions (13 h light/11 h dark, 20°/16°C) to induce flowering.

### Chromosome preparation

Mitotic metaphase accumulation and fixation procedures were carried out according to Kwiatek et al. (2016a). Enzymatic digestion was made in 0.2% (v/v) Onozuka R-10 and Calbiochem cytohelicases (1:1 ratio) and 20% pectinase (Sigma) in 10 mM citrate buffer (pH 4.6) at 37°C for 2 h and 40 min and stopped by washing twice, for 10 min each, in citrate buffer. Chromosome preparations were made according to the protocol reported by Heckmann et al. (2014) with a minor modification. Root tips were placed in depression slides in ice-cold 60% acetic acid. Apical meristems were separated from the rest of root tips by using needles and were placed on slides in a drop of ice-cold 60% acetic acid and dispersed with a metal needle. The slides were heated on a heating table (Medax) for 2 min at 48°C. Afterwards, the slides were removed from the hot plate and 200 μl of ice-cold ethanol-acetic acid (3:1, v/v) was added to wash the slides briefly. Then the slides were placed in 60% acetic acid for 10 min, followed by washing in 96% ethanol and air dried.

Meiotic chromosome spreads of macerated anthers were prepared from flower buds fixed with ethanol-acetic acid (3:1, v/v) according to Zwierzykowski et al. (2008) with minor modifications. Anthers were placed onto a slide in a drop of ice-cold 60% acetic acid and dispersed with a metal needle. Ice-cold acetic acid (60%) was added to the cell suspension. The slide was placed on a hot plate (45°C) for 2 min. During this time, the drop was stirred by the needle. The slide was removed from the hot plate and freshly prepared ice-cold ethanol–acetic acid (3:1, v/v) was added and covered by the cover slip. After quick freezing in liquid nitrogen, the cover slip was removed.

### Lagging chromosomes

The mean numbers of lagging chromosomes (divided into *Secale* and *Triticum* genomes) at anaphase II of PMCs of four monosomic 4M^g^ addition plants of triticale were calculated considering 50 cells of each plant and compared between cells carrying/lacking the 4M^g^ chromosome, by using analysis of variance (ANOVA) and Tukey's Honest Significant Difference (HSD) test at *P* = 0.05 and *P* = 0.01 significance levels (STATISTICA 13.1, StatSoft Poland).

### Probe preparation and fluorescence *in situ* hybridization

Total genomic DNA was isolated using DNeasy Plant Maxi Kit 24 (Qiagen). DNA of *Aegilops comosa* Sm. (PI 551020, U.S. National Plant Germplasm System), a progenitor of the M-genome of *Ae. geniculata*, was labeled by nick translation with Atto-488 dye (Atto-488NT kit; Jena Bioscience) for investigation of 4M^g^ chromosome behavior during meiotic divisions of PMCs of M4M^g^A triticale. Total genomic DNA of rye (Imperial) was labeled in the same way by Atto-550 dye. Blocking DNA from *T. durum* (AABB, Duromax) or triticale (AABBRR, Moreno) was sheared by boiling for 30–45 min and used at a ratio of 1:50 (probe:block). Genomic *in situ* hybridization (GISH) or multicolor GISH was performed according to previously published protocols (Kwiatek et al., 2016a,c). Mitotic chromosomes were identified using fluorescence *in situ* hybridization (FISH) with the repetitive sequences from pTa-86, pTa-103, and pTa-535 clones as well as GAA microsatellite characterized by Komuro et al. (2013), amplified from genomic DNA of wheat (Chinese Spring) according to Kwiatek et al. (2016b), and labeled by nick translation kits (Jena Bioscience) using Atto-488, Atto-550, and Atto-647 dyes. FISH was performed according to Kwiatek et al. (2016b). Slides were examined with an Olympus XM10 CCD camera attached to an Olympus BX 61 automatic epifluorescence microscope. Image processing was carried out using Olympus Cell-F (version 3.1; Olympus Soft Imaging Solutions GmbH: Münster, Germany) imaging software and PaintShop Pro X5 software (version 15.0.0.183; Corel Corporation, Ottawa, Canada). Particular chromosomes were identified by comparing the signal patterns of the probes (Komuro et al., 2013; Kwiatek et al., 2013, 2016b).

### Anther cultures and DH production

Tillers from M4M^g^A triticale plants were cut at the microspore stage and treated in mineral salt medium N6 (Chu, 1975) supplied with 2,4-D (2.0 mg/l) in the dark at 4°C for 6–9 days. Anthers were cultured on the C17 medium (Wang and Chen, 1983) with 2,4-D (2.0 mg/l), kinetin (KIN, 0.5 mg/l), and maltose (90 g/l) in darkness at 28°C. Androgenic structures were placed in regeneration medium 190-2 (Zhuang and Xu, 1983) at 22°C, in continuous light for 12 h per day. Ploidy levels of the androgenic and regenerated plants were evaluated by fluorescence (DAPI) using a Partec II (Sysmex Partec GmbH, Germany) flow cytometer (de Laat et al., 1987). Leaf fragments were chopped with a sharp razor blade in a Petri dish with 2 ml of the nuclei-isolation buffer [0.1 M Tris, 2.5 mM MgCl_2_·6H_2_O, 85 mM NaCl, 0.1% (v/v) Triton X-100] containing DAPI and the cell suspension was filtered through a 30-μm filter. Plants containing 1C DNA were identified as haploids, while those with 2C DNA, as diploids. Chromosome doubling was conducted using 0.1% colchicine solution, with DMSO (4%) and GA3 supplement (25 mg/l). Finally, the plants were placed in a growth chamber at 4°C for 6 weeks and then located in the greenhouse until harvest.

### Simple sequence repeat (SSR) marker analysis

Genomic DNA of 10 plants of each DH line was isolated using Plant DNA Purification Kit (EurX Ltd., Poland). DNA quality and quantity were determined using 1% agarose gel and a spectrophotometer. DNA concentration was adjusted to 50 ng/μL. Sequences of 30 wheat-specific SSR markers were amplified using specific primers (GrainGenes 2.0, **Table 5**) via polymerase chain reaction (PCR). The PCR reaction volume was 20 μL, consisting of 150 nM each of the 2 primers, 0.2 mM each of the nucleotides, 1.5 mM MgCl_2_, 0.2 units of Taq-DNA hot-start polymerase (Xceed, Biotech-Poland), and 50 ng of genomic DNA as a template. A typical PCR procedure was as follows: 5 min at 95°C, then 35 cycles of 30 s at 94°C, 30 s at 50–60°C (depending on the primer), 1 min at 72°C, and 5 min at 72°C. Low-copy and high-quality SSR markers with relatively high polymorphic information content were selected based on data provided by the GrainGenes database (GrainGenes2.0 2016). PCR products were run on 3% agarose gel (Sigma) with 1% TBE buffer. DNA was visualized via Midori Green Direct (Nippon Genetics Europe) added to the samples.

## Results

### Meiotic analysis of monosomic 4M^g^ addition hexaploid triticale plants

**Figure 1:**
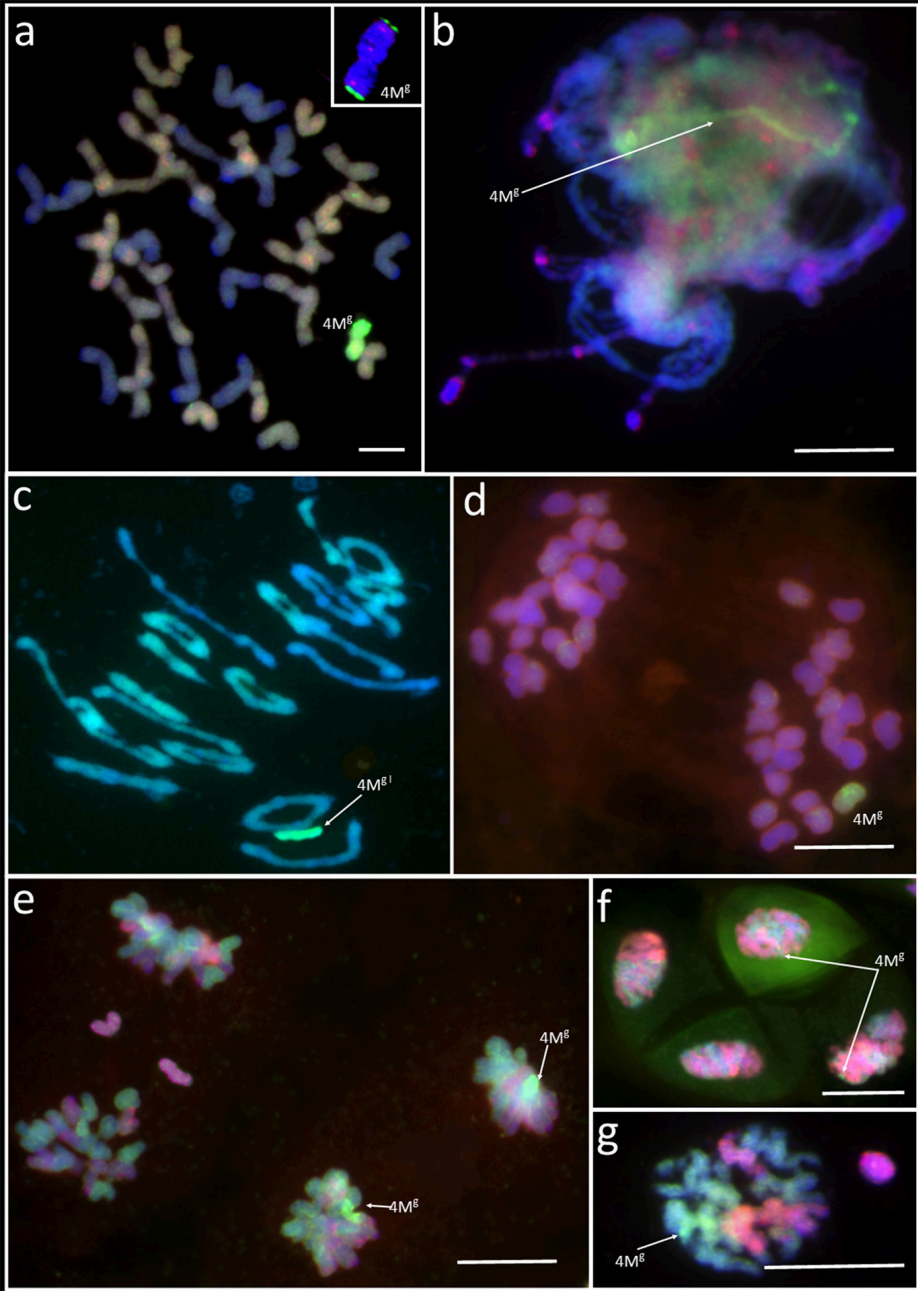
**Genomic *in situ* hybridization (GISH) with genomic DNA of *Aegilops comosa* (MM) labeled with Atto647 (green) identifies chromosome 4M^g^ in a monosomic addition plant of triticale (×*Triticosecale*) (a)** at metaphase of mitosis in the root apical meristem; **(b)** at prophase I (pachytene) of meiosis; **(c)** metaphase I of meiosis; **(d)** anaphase/telophase I of meiosis; **(e)** anaphase/telophase II of meiosis; **(f,g)** telophase/pollen tetrad stage of meiosis of pollen mother cells. Arrows show 4M^g^ chromatin/chromosome. The inset in **(a)** shows chromosome 4M^g^ identified using fluorescence *in situ* hybridization (FISH) with pTa-86 and pTa-535 probes. Scale bar: 10 μm.

Four monosomic addition hexaploid triticale plants carrying a 4M^g^ chromosome from *Aegilops geniculata* (M4M^g^A) (Figure 1a) were selected from the offspring of F_2_BC_1_triticale × *Ae. geniculata* hybrids, which were previously generated by Kwiatek et al. (2016c). A GISH analysis of meiosis of PMCs showed that chromosome 4M^g^ was present as a univalent at prophase I (Figure 1b) and metaphase I of meiosis (Figure 1c). M4M^g^A plants had no meiotic irregularities and chromosomes paired in the form of 21 bivalents plus a 4M^g^ univalent (Figure 1c) or as 20 bivalents plus three univalents, including a 4M^g^ chromosome (Table 1). Chromosome 4M^g^ was transmitted into one of two daughter cells formed after the first cell division (Meiosis I; Figure 1d). Furthermore, sister chromatids of this chromosome were separated to opposite cell poles during the second cell division. During anaphase II we observed 1-3 lagging chromosomes of triticale (Figure 1e). The ANOVA showed no significant differences between the four M4M^g^A plants. The mean number of lagging chromosomes was 0.11 in the daughter cells carrying the 4M^g^ chromosome, and 0.76 in the daughter cells lacking 4M^g^. Interestingly, the Tukey's highest significant difference test revealed that the frequency of lagging R-genome chromosomes was significantly higher than the frequency of A- or B- genome chromosomes in each plant (Table 2). Additionally, micronuclei were observed in 3.85% of pollen tetrads and carried only R-genome chromatin (Figures 1f,g). Finally, the mean pollen viability of M4M^g^A plants was 35.9% and ranged between 29.3 and 48.0%.

**Table 1:** **Genomic *in situ* hybridization (GISH) analysis of specificity of chromosome pairing (in bivalents) in four monosomic addition hexaploid plants of triticale (×*Triticosecale*) carrying a 4M^g^ chromosome from *Aegilops geniculata* (M4M^g^A)**.

**Cell no**	**No. of cells**	**Mean no. of univalents per cell**			**Mean no. of bivalents per cell**		
		**Total**	***Ae. geniculata***	**Triticale**	**Triticale**		
					**Total**	**Ring**	**Rod**
MA4M^g^_1	30	1.27	1.00	0.21	20.87	12.43	8.43
MA4M^g^_2	30	1.20	1.00	0.20	20.90	12.60	8.30
MA4M^g^_3	30	1.13	1.00	0.13	20.93	12.77	8.17
MA4M^g^_4	30	1.13	1.00	0.13	20.93	12.70	8.23

**Table 2:** **Mean number and range of lagging chromosomes at anaphase II of pollen mother cells of four monosomic 4M^g^ addition plants of triticale (×*Triticosecale*) studied in 50 cells per plant**.

	**MA4M^g^_1**				**MA4M^g^_2**				**MA4M^g^_3**				**MA4M^g^_4**			
	**4M^g^+**		**4M^g^-**		**4M^g^+**		**4M^g^-**		**4M^g^+**		**4M^g^-**		**4M^g^+**		**4M^g^-**	
	**1**	**2**	**3**	**4**	**1**	**2**	**3**	**4**	**1**	**2**	**3**	**4**	**1**	**2**	**3**	**4**
	**AB**	**R**	**AB**	**R**	**AB**	**R**	**AB**	**R**	**AB**	**R**	**AB**	**R**	**AB**	**R**	**AB**	**R**
**Mean (range)**	**0.1 (0–1)**	**0.16 (0–1)**	**0.26 (0–2)**	**1.02 (0–3)**	**0.08 (0–1)**	**0.12 (0–1)**	**0.18 (0–2)**	**1.34 (0–3)**	**0.06 (0–1)**	**0.14 (0–1)**	**0.28 (0–2)**	**1.36 (0–3)**	**0.04 (0–1)**	**0.16 (0–1)**	**0.36 (0–2)**	**1.24 (0–3)**
Variance	0.091837	0.137143	0.237143	0.79551	0.075102	0.107755	0.191429	0.718776	0.057551	0.122857	0.328163	0.765714	0.039184	0.137143	0.398367	0.635102
Std. Dev.	0.303046	0.370328	0.486973	0.891914	0.274048	0.328261	0.437526	0.847806	0.239898	0.35051	0.572855	0.875051	0.197949	0.370328	0.631163	0.796933
Std. Err.	0.042857	0.052372	0.068868	0.126136	0.038756	0.046423	0.061875	0.119898	0.033927	0.04957	0.081014	0.123751	0.027994	0.052372	0.08926	0.112703
HSD 0.05	0.29				0.27				0.29				0.29			
HSD 0.01	0.35				0.33				0.36				0.35			
1 vs 2	nonsignificant				nonsignificant				nonsignificant				nonsignificant			
1 vs 3	nonsignificant				nonsignificant				nonsignificant				*P* < 0.05			
1 vs 4	*P* < 0.01				*P* < 0.01				*P* < 0.01				*P* < 0.01			
2 vs 3	nonsignificant				nonsignificant				nonsignificant				nonsignificant			
2 vs 4	*P* < 0.01				*P* < 0.01				*P* < 0.01				*P* < 0.01			
3 vs 4	*P* < 0.01				*P* < 0.01				*P* < 0.01				*P* < 0.01			

*4M+/4M− = cells carrying/lacking 4M^g^ chromosome; AB = chromosomes of Triticum genomes; R = chromosomes of Secale genome; Std. Dev. = standard deviation; Std. Err. = standard error; HSD 0.05/0.01 = Tukey's highest significant difference tested at P = 0.05 and P = 0.01 significance levels*.

### Production of DH lines

**Figure 2:**
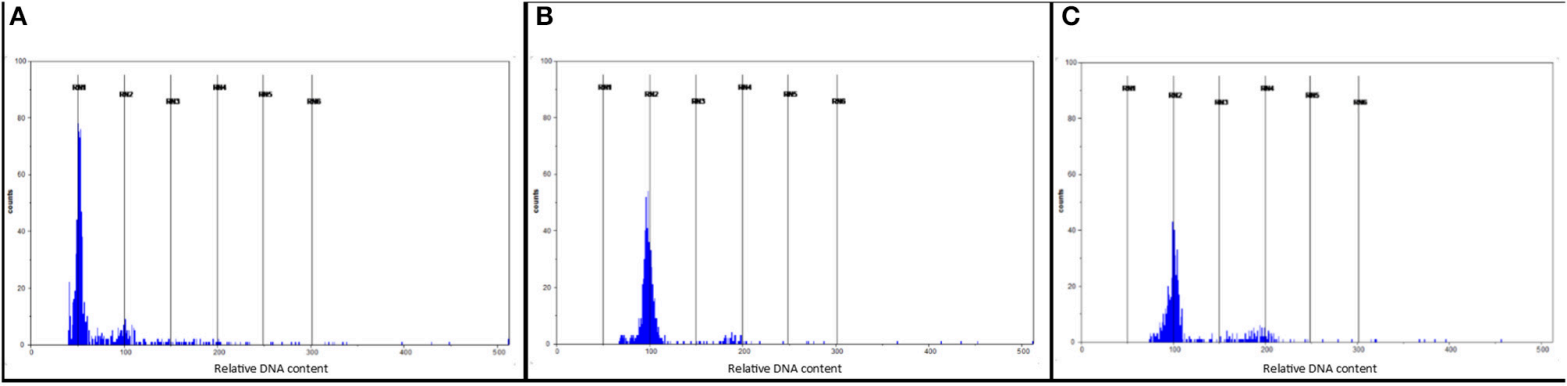
**Flow cytometry histograms showing (A)** haploid plants (in channel 50); **(B)** doubled haploid (DH) plants of triticale (×*Triticosecale*) after colchicine treatment (in channel 100) obtained from monosomic addition hexaploid triticale plants carrying a 4M^g^ chromosome from *Aegilops geniculata* (M4M^g^A) and **(C)** control triticale variety “Moreno” (in channel 100). Nuclei released from haploid plants contained only C DNA, while those released from DH plants contained 2C DNA.

A total of 4,313 anthers collected from four M4M^g^A plants were used to produce 4,373 androgenic structures. The highest number of androgenic structures (2,538) was obtained from the anthers of plant M4M^g^A_3, whereas the lowest number of those structures (183) was obtained from anthers of plant M4M^g^A_1. Overall, 77 haploid plants were obtained, of which 42 were generated from plant M4M^g^A_1. Finally, 55 DH plants were generated and 41 of them were self-pollinated and harvested. Flow cytometry experiments confirmed the ploidy level of androgenic plants, derived DH plants and control triticale variety “Moreno” (Figures 2A–C, respectively). The efficiency of androgenesis and numbers of spikes and seeds of DH lines are presented in Tables 3, 4, respectively.

**Table 3:** **Effectiveness of androgenesis induction and doubled haploid production from four monosomic 4M^g^ addition plants of triticale (×*Triticosecale*)**.

**Plant**	**Number of anthers**	**Androgenic structures**		**Green plants**		**Plants for colchicine treatment**	**Haploids**		**Doubled haploids**		**Aneuploids**	
		**Number**	**Per 100 anthers**	**Number**	**Per 100 anthers**		**Number**	**%**	**Number**	**%**	**Number**	**%**
M4M^g^A_1	950	183	19.3	42	4.4	42	16	38.1	26	61.9	0	0
M4M^g^A_2	1020	1450	142.2	12	1.2	10	1	10	9	90	0	0
M4M^g^A_3	1386	2538	183.1	16	1.2	13	0	0	13	100	0	0
M4M^g^A_4	957	1202	125.6	7	0.7	7	0	0	7	100	0	0

**Table 4:** **Characterization of doubled haploid (DH) lines obtained by anther cultures from four monosomic 4M^g^ addition plants of triticale (×*Triticosecale*)**.

**Plant**	**DH lines**						
	**Total number**	**Chromosome constitution**	**Number of lines**	**%**	**Number of spikes**	**Number of seeds**	**Number of analyzed plants**
M4M^g^A_1	13	40T + Dt2RS + Dt2RL	5	38.5	68	4827	50
		42T + M4Mg	7	53.9	84	5523	70
		40T + N2R	1	7.6	12	835	10
M4M^g^A_2	8	38T + D4RS.4BL	3	37.5	37	2563	30
		42T + D4Mg	5	62.5	61	3825	50
M4M^g^A_3	13	38T + D5BS-5BL.5RL	5	38.5	67	4197	50
		40T + D4Mg	8	61.5	92	5846	80
M4M^g^A_4	7	38T + D3AS.7RL	3	42.9	41	2763	30
		42T + D4Mg	4	57.1	59	3751	40

### Karyotyping of the DH plants

**Figure 3:**
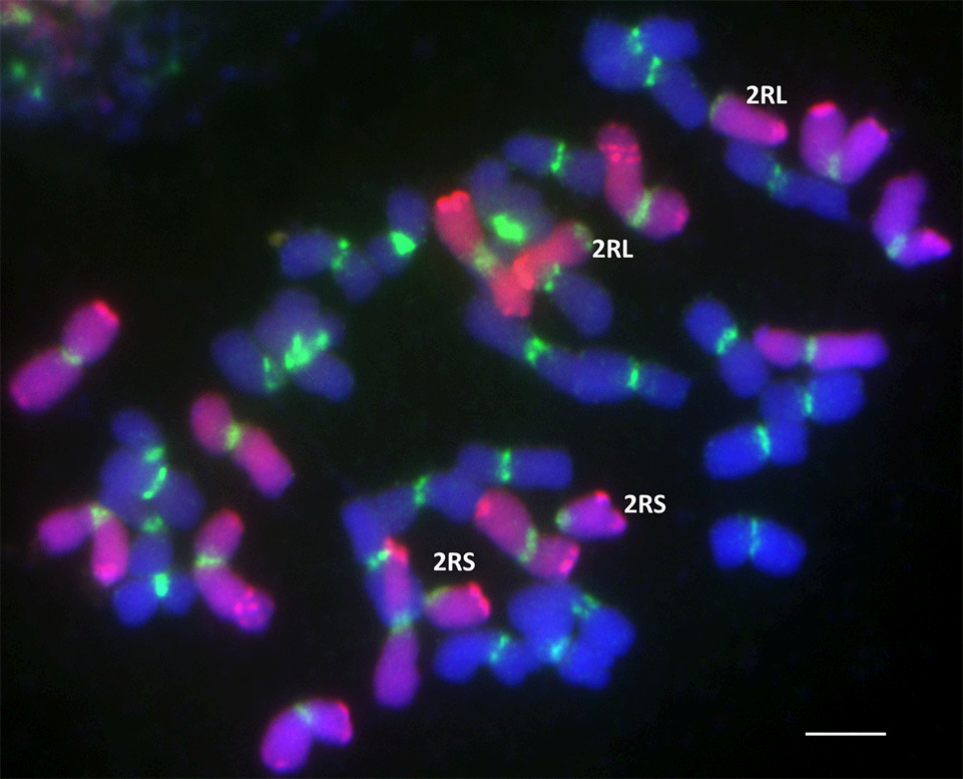
**Genomic/Fluorescence *in situ* hybridization (GISH/FISH) with genomic DNA probe of *Secale cereale* (R-genome chromosomes; Atto550; red) and centromere pTa-103 probe (Atto488; green) blocked with genomic DNA of *Triticum durum* (A- and B-genome chromosomes; DAPI; blue) showing two pairs of telocentric chromosomes 2RS and 2RL in 40T+Dt2RS+Dt2RL doubled haploid plants of triticale (×*Triticosecale*)**. Scale bar: 10 μm.

**Figure 4:**
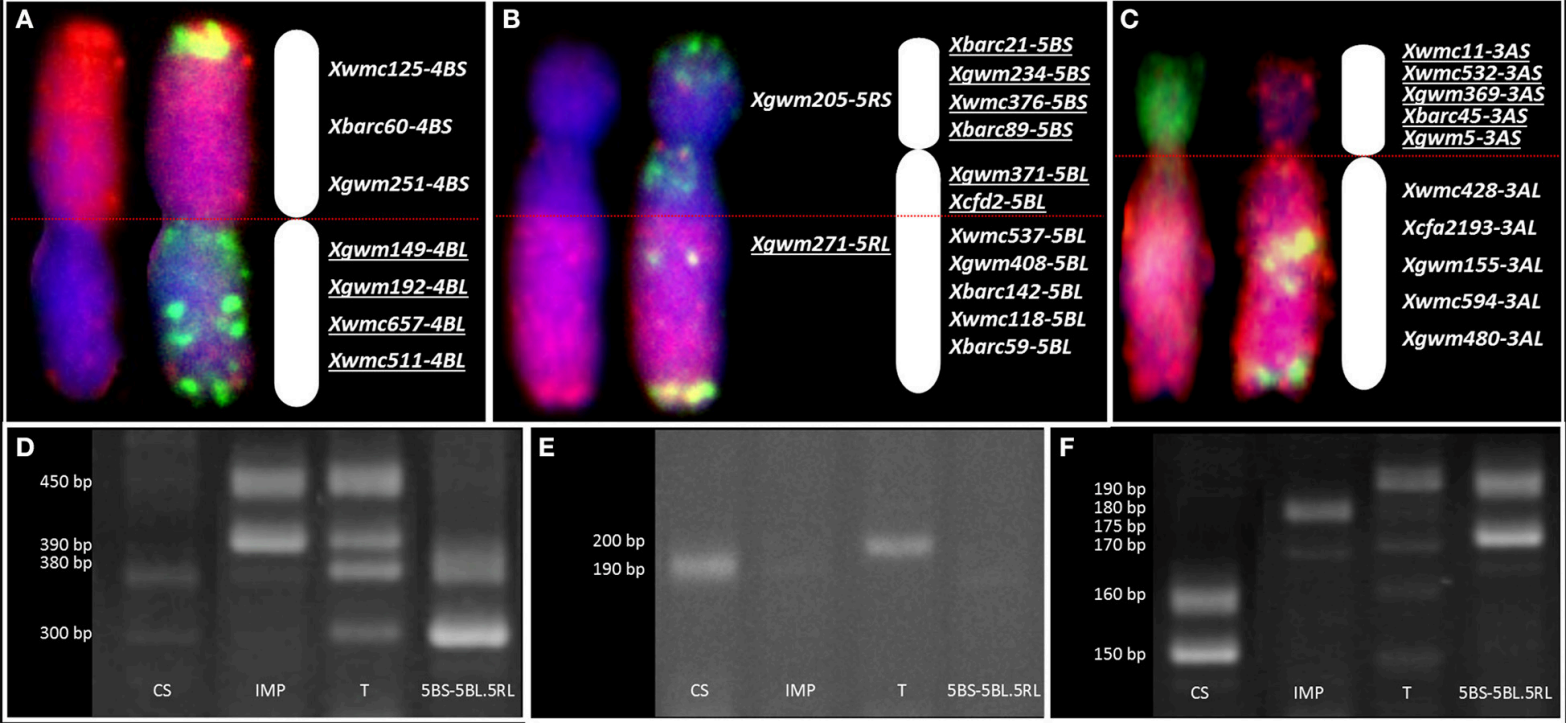
**Physical and genetic mapping correlation. Genomic *in situ* hybridization (GISH) pattern, fluorescence *in situ* hybridization (FISH) with pTa-86+GAA (Atto488; green) and pTa-535 (Atto550; red), and wheat-specific SSR markers mapped on (A)** 4RS.4BL; **(B)** 5BS-5BL.5RL and **(C)** 3AS.7RL translocation chromosomes of triticale (×*Triticosecale*). GISH analysis was made using genomic probes of *Triticum urartu* (Atto488; green), *Secale cereale* (Atto-550, red), and blocking DNA of *Aegilops speltoides* (DAPI, blue) to distinguish A-, R-, and B-genome triticale chromosomes, respectively. Underlined markers indicate the presence of a particular chromosome segment. Band patterns obtained for the wheat SSR markers **(D)** *Xcfd2-5BL*, **(E)** *Xwmc537-5BL*, and rye SSR marker **(F)** *Xgwm271-5RL* showing the genetic region of 5BL.5RL translocation. (CS) wheat “Chinese Spring”—control sample, (IMP) rye “Imperial”—control sample, (T) triticale “Moreno”—control sample and (5BS-5BL.5RL) 38T+D5BS-5BL.5RL DH plant bearing a pair of 5B chromosomes with a segment of long arm of 5R chromosome.

Root meristems from ten seeds of each of 41 DH lines were randomly chosen for karyotyping using FISH and GISH. In each case, 10–20 cells were studied. The chromosome constitutions and the number of related DH lines are presented in Table 4. The karyotype analysis revealed that 24 of 41 DH lines (58.54%) possessed a complete set of triticale chromosomes (2*n* = 6*x* = 42; AABBRR) and chromosome 4M^g^. The other 17 lines carried chromosome aberrations and no chromosome 4M^g^. From plant MA4M^g^_1, we obtained one DH line, where the plants had 40 chromosomes without 2R chromosome pair (40T+N2R). In plants of five other DH lines, we identified four telocentric chromosomes consisting of two 2RS and two 2RL chromosome arms (40T+Dt2RS+Dt2RL, Figure 3). This shows that the gametocidal action resulted in fragmentation of chromosome 2R. Plant MA4M^g^_2 was a donor of three DH lines of triticale plants, which carried a pair of translocation chromosomes built out of a short arm of chromosome 4R and a long arm of chromosome 4B (38T+4RS.4BL, Figure 4A). Moreover, complete 4R and 4B chromosomes were not observed, indicating that chromosomes 4B and 4R were fragmented and remodeled as translocation chromosomes. Plants of five DH lines originated from plant MA4M^g^_3, which carried 40 chromosomes, including a pair of chromosomes 5B with a translocation of a segment of the long arm from chromosome 5R (38T+D5BS-5BL.5RL). Moreover, complete 5B and 5R chromosomes were absent in those plants (Figure 4B). All chromosomes 5B and 5R were damaged by the gametocidal action and remodeled, as a consequence. From plant MA4M^g^_4, we obtained three DH lines containing plants with 38 chromosomes, including a pair of chromosomes containing a short arm of chromosome 3A and a long arm of chromosome 7R (3AS.7RL) and no complete 3A and 7R chromosomes (38T+D3AS.7RL, Figure 4C).

### PCR analysis of chromosome breakpoint loci

We used 30 wheat-specific SSR markers to screen the breakpoint loci of rearranged chromosomes in 10 plants of each of the DH translocation lines (Table 5). Markers were chosen to cover both short and long arms of the A- or B-genome (*Triticum*) chromosomes involved in translocation with R-genome (*Secale*) chromosome segments. Three SSR markers linked to the 4BS chromosome arm showed its presence (Figure 4A). The amplification of marker *Xbarc60* resulted in two products that allowed us to differentiate between 4BS and 4RS chromosome arms. For mapping of chromosome 3A we have chosen 10 SSR markers (five markers for the short arm and five for the long one). Two or more products of amplification were observed in case of three markers (*Xbarc45-3AS, Xgwm5-3AS*, and *Xwmc428-3AL*). Besides, the amplification products of nine markers (except *Xwmc532*-3AS) were different when comparing control varieties of wheat (Chinese Spring) and rye (Imperial) with triticale (Moreno). In order to identify 5B chromosome segments, we used 11 markers linked to both short and long arms of this chromosome (Figure 4B). The SSR analysis showed that four markers linked to the short arm, while two markers connected with the long arm (*Xcfd2-5BL* and *Xwmc537-5BL*, Figures 4D,E, respectively) were identified in 38T+D5BS-5BL.5RL, indicating that the 5B chromosome segment includes the centromere. Additional SSR analysis with *Xgwm271-5RL* (Figure 4F) and *Xgwm6-5RL* markers showed that the 5RL.5BL breakpoint locus is placed between *Xcfd2-5BL* and *Xgwm271-5RL* loci. A similar approach was used to map 3A chromosome segments in 38T+D3AS.7RL DH translocation plants. All five markers proved the presence of the short arm of the 3A chromosome in the 38T+D3AS.7RL plants, while markers linked to the 3AL chromosome indicated a lack of this arm (Figure 4C).

**Table 5:** **Analysis of simple sequence repeat (SSR) markers specific to chromosomes involved in intergenomic chromosome translocations in doubled haploid (DH) plants of triticale (×*Triticosecale*)**.

**No**.	**SSR marker**	**Chromosome arm**	**Primer sequences (5′→3′)**		**Amplification products (bp)**					
					**Control genotypes**			**DH lines with *Triticum/Secale* chromosome translocations**		
			**Forward**	**Reverse**	***Triticum aestivum* “Chinese Spring”**	***Secale cereale* “Imperial”**	**Triticale “Moreno”**	**38T+D3AS.7RL**	**38T+D4RS.4BL**	**38T+D5BS-5BL.5RL**
1	*Xwmc11*	3AS	TTGTGATCCTGGTTGTGTTGTGA	CACCCAGCCGTTATATATGTTGA	180	0	185	185	n/a	n/a
2	*Xwmc532*	3AS	GATACATCAAGATCGTGCCAAA	GGGAGAAATCATTAACGAAGGG	180	0	180	180	n/a	n/a
3	*Xgwm369*	3AS	CTGCAGGCCATGATGATG	ACCGTGGGTGTTGTGAGC	190	280	200 + 280	200	n/a	n/a
4	*Xbarc45*	3AS	CCCAGATGCAATGAAACCACAAT	GCGTAGAACTGAAGCGTAAAATTA	170 + 180	0	190 + 200	190 + 200	n/a	n/a
5	*Xgwm5*	3AS	GCCAGCTACCTCGATACAACTC	AGAAAGGGCCAGGCTAGTAGT	180 + 190	280	180 + 200 + 290	180 + 200	n/a	n/a
6	*Xwmc428*	3AL	TTAATCCTAGCCGTCCCTTTTT	CGACCTTCGTTGGTTATTTGTG	260 + 270	0	280 + 300	0	n/a	n/a
7	*Xcfa2193*	3AL	ACATGTGATGTGCGGTCATT	TCCTCAGAACCCCATTCTTG	210	0	220	0	n/a	n/a
8	*Xgwm155*	3AL	CAATCATTTCCCCCTCCC	AATCATTGGAAATCCATATGCC	140	0	150	0	n/a	n/a
9	*Xwmc594*	3AL	TGTGCGGAATGATTCAATCTGT	GGCCATTAGACTGCAATGGTTT	180	0	200	0	n/a	n/a
10	*Xgwm480*	3AL	TGCTGCTACTTGTACAGAGGAC	CCGAATTGTCCGCCATAG	200	0	210	0	n/a	n/a
11	*Xwmc125*	4BS	ATACCACCATGCATGTGGAAGT	ACCGCTTGTCATTTCCTTCTGT	260	0	260	n/a	0	n/a
12	*Xbarc60*	4BS	CATGCTCACAAAACCCACAAGACT	CTCGAAAGGCGGCACCACTA	225	200	200 + 230	n/a	200	n/a
13	*Xgwm251*	4BS	CAACTGGTTGCTACACAAGCA	GGGATGTCTGTTCCATCTTAG	110	0	100	n/a	0	n/a
14	*Xgwm149*	4BL	CATTGTTTTCTGCCTCTAGCC	CTAGCATCGAACCTGAACAAG	170	0	170	n/a	170	n/a
15	*Xgwm192*	4BL	GGTTTTCTTTCAGATTGCGC	CGTTGTCTAATCTTGCCTTGC	190	0	200	n/a	200	n/a
16	*Xwmc657*	4BL	CGGGCTGCGGGGGTAT	CGGTTGGGTCATTTGTCTCA	130	0	130	n/a	130	n/a
17	*Xwmc511*	4BL	CGCACTCGCATGATTTTCCT	ATGCCCGGAAACGAGACTGT	200	0	200	n/a	200	n/a
18	*Xbarc21*	5BS	GCGTCTTCCGGTTTTGTTTACTTTTC	GCGTTAGGGCTATGGCGGTGTG	220	470	220 + 470	n/a	n/a	220
19	*Xgwm234*	5BS	GAGTCCTGATGTGAAGCTGTTG	CTCATTGGGGTGTGTACGTG	200 + 240	350	200 + 250 + 350	n/a	n/a	200 + 250
20	*Xwmc376*	5BS	TCTCAACCACCGACTTGTAA	ACATGTAATTGGGGACACTG	210	0	210	n/a	n/a	210
21	*Xbarc89*	5BS	GGGCGCGGCACCAGCACTACC	CTCCGAGGCCACCGAAGACAAGATG	130	0	140	n/a	n/a	140
22	*Xgwm371*	5BL	GACCAAGATATTCAAACTGGCC	AGCTCAGCTTGCTTGGTACC	170	0	170	n/a	n/a	170
23	*Xcfd2*	5BL	GGTTGCAGTTTCCACCTTGT	CATCTATTGCCAAAATCGCA	300 + 380	390 + 450	300 + 380 + 390 + 450	n/a	n/a	300 + 380
24	*Xwmc537*	5BL	TCTTCTGTACATTGAACAACGA	ATGCAGAACCGTGATAGGAT	190	0	200	n/a	n/a	0
25	*Xgwm408*	5BL	TCGATTTATTTGGGCCACTG	GTATAATTCGTTCACAGCACGC	180	0	185	n/a	n/a	0
26	*Xbarc142*	5BL	CCGGTGAGAGGACTAAAA	GGCCTGTCAATTATGAGC	270	240	230 + 265	n/a	n/a	230
27	*Xwmc118*	5BL	AGAATTAGCCCTTGAGTTGGTC	CTCCCATCGCTAAAGATGGTAT	140	0	135	n/a	n/a	0
28	*Xbarc59*	5BL	GCGTTGGCTAATCATCGTTCCTTC	AGCACCCTACCCAGCGTCAGTCAAT	170	0	175	n/a	n/a	0
29	*Xgwm205*	5RS	CGACCCGGTTCACTTCAG	AGTCGCCGTTGTATAGTGCC	140 + 150	130	130 + 140 + 150	n/a	n/a	140 + 150
30	*Xgwm271*	5RL	CAAGATCGTGGAGCCAGC	AGCTGCTAGCTTTTGGGACA	150 + 160	170 + 180	155 + 160 + 175 + 190	n/a	n/a	175 + 190

*n/a = not applicable*.

## Discussion

Until now, *Aegilops*-specific Gc factors were identified in S-genome, C-genome, and M-genome species (Endo, 2007). All of the already identified Gc factors are located on homeologous groups 2, 3, 4, and 6 chromosomes of *Aegilops* species (Endo, 2007). Previous studies showed that Gc genes, like other pollen killer factors, ensure their preferential transmission by inducing chromosome breakage in gametophytes lacking them (Nasuda et al., 2005a). The induced chromosome breakage occurred after the meiotic divisions of PMCs and took place in the interphase before the first pollen mitosis (Nasuda et al., 1998). The 4M^g^ gametocidal chromosome action is the least studied. Hence, in this study we used monosomic addition plants of hexaploid triticale (AABBRR) carrying the 4M^g^ chromosome from *Ae. geniculata* to induce the chromosome rearrangements between the A- or B-genome (*Triticum*) and the R-genome (*Secale*) chromosomes of triticale. Kynast et al. (2000) first reported that a Gc factor is present on group 4 chromosomes of the M-genome of *Ae. geniculata*, which was analyzed using wheat (Chinese Spring) monosomic 4M^g^ addition plants. Our research team has presented its preferential transmission and gametocidal properties in triticale (Kwiatek et al., 2016c). An investigation of monosomic *Triticum aestivum-Ae. geniculata* chromosome 4M^g^ addition plants revealed that male gametogenesis in disomic 4M^g^ addition plants is normal (Kynast et al., 2000). However, chromosome breakage and anaphase bridges were observed at anaphase and/or telophase of the first and second pollen mitosis. In the present study, the investigation of the first (MI) and second (MII) meiotic divisions of PMCs of triticale monosomic 4M^g^ addition plants shows the regular transmission of the 4M^g^ univalent to one of two daughter cells (in MI), giving rise to four haploid cells, and half of them carried a 4M^g^ chromosome (MII). In contrast to previous studies, we have observed lagging chromosomes at anaphase II of PMCs. Intriguingly, the number and frequency of those chromosomes were significantly higher in the cells lacking a 4M^g^ chromosome, in comparison to cells containing this gametocidal chromosome. This may indicate that the gametocidal action is initiated right at the second meiotic division of daughter cells lacking the Gc chromosome. It is possible that the Gc factor induced a higher number and frequency of chromosome lagging, as well. Such developments can have multiple fates. Lagged chromosomes can be trapped in the cytokinetic furrow and break during cytokinesis. Alternatively, they can form their own micronucleus, which is either accurately segregated or mis-segregated (Santaguida and Amon, 2015). Irrespective of how the broken chromosomes are segregated, their segments can be incorporated into other chromosomes. Moreover, it could be hypothesized that early appearance of gametocidal action could be related to the lack of Gc suppressor gene in triticale (Moreno) or its incomplete form. Friebe et al. (2003) showed that Gc genes encode two factors: one causing chromosome breakage in gametophytes lacking them, and another factor protecting the gametophytes that have the Gc-carrier chromosome. Both the Gc gene and the suppressor are located on chromosomes of the same homeologous group (Tsujimoto, 2005). Moreover, some cultivars of common wheat carry a gene that suppresses the function of the Gc gene, but some others (i.e., “Chinese Spring”) carry an incomplete suppressor, which makes pollen without the Gc chromosome functional and transmitted to the progeny (Tsujimoto, 2005).

We induced the androgenesis of M4M^g^A triticale using anther cultures for further evaluation of the 4M^g^ gametocidal action. However, our main goal was to catch the possible chromosomal rearrangements and maintain them by the generation of DHs. The induction of androgenesis usually starts with the production of embryo-like structures via the development of microspores. To achieve this, the normal development of microspores has to be blocked and turned toward sporophytic development. This usually takes place during the first pollen mitosis. Hence, this process led us to obtain haploid plants from both microspores carrying the Gc chromosome (4M^g^) and microspores lacking it. The number of DH lines with the doubled 4M^g^ chromosome was 24 (58.54%), while the number of DH lines without the Gc chromosome, carrying chromosome aberrations, was slightly lower (17; 41.46%). In other words, despite the gametocidal action, which appeared during anaphase II of meiosis in the cells lacking the Gc chromosome, we have obtained 17 of 41 DH lines with chromosome rearrangements. Of course, the induced androgenesis via anther cultures itself can provide cytogenetic abnormalities, including ploidy changes and chromosome rearrangements (Kaeppler et al., 2000). Moreover, triticale is a less meiotically stable crop, compared to wheat, and that is manifested by chromosome pairing issues and tends to show a fair proportion of aneuploids among both sexually derived progenies and DH lines (Lukaszewski and Gustafson, 1987; Lelley, 2006). Oleszczuk et al. (2011) karyotyped (not randomly, basing on morphology) 140 DH plants of winter triticale and observed some plants with chromosome abnormalities, such as different chromosome number (nullisomics and tetrasomics), telocentric chromosomes, and translocations. What is more, most of the plants were nullisomics for one or more rye chromosomes, and chromosome 5R was the most frequently involved, followed by 2R. However, those chromosomal abnormalities were not constant, which means that different types of chromosome aberrations were observed, considering individual DH lines. In contrast, our study shows that the karyotypes of 10 plants from each of the 41 DH lines were identical, so apparently the chromosome set was established during meiosis of PMCs by the gametocidal action and has not been changed during androgenesis. Interestingly, chromosomes 2R and 5R were involved in chromosome translocations. Molecular cytology and SSR marker analysis enabled us to confirm the uniformity of particular DH lines in terms of their karyotypes and to identify the breakpoint loci in the translocation forms. In case of 40T+Dt2RS+Dt2RL (five lines), 38T+D4RS.4BL (three lines), and 38T+D7RS.3AL (three) plants, the breakpoints were located in the centromere. Molecular analysis of 38T+D5BS-5BL.5RL (five lines) plants showed that the chromosome breakage took place between *Xcfd2* and *Xwmc537* loci on chromosome 5B and the distal part of a long arm of chromosome 5R carrying *Xgwm271-5RL* and *Xgwm6-5RL* markers. From the other point of view, the gametocidal action contributed to elimination of triticale chromosome arms (3AS, 3RS, 4RL, 7RL) and segments (distal segment of 5BL and 5RS-5RL.del). Intriguingly, some of the reports indicated that chromosome 5R is involved in induction of chromosome abnormalities (Charmet et al., 1986; Oleszczuk et al., 2011). It is also worth underlining that chromosome 5B carries the *Ph1* gene locus (5BL), which enforces strictly bivalent pairing in polyploid wheat, and this makes 38T+D5BS-5BL.5RL DH lines a great germplasm for meiotic studies.

In summary, this approach proves that gametocidal action can be applied to induce intergenomic chromosome aberrations in triticale. Besides, the application of this mechanism in combination with anther culture induction, followed by DH line production, revealed a sufficiently large population of homozygous DH individuals with two identical copies of each chromosome, especially translocated ones. This system seems to be fit for breeding programs to produce homozygous forms in terms of particular chromosome rearrangements. Those forms are ideal for genetic mapping and can be used for the development of high-density marker maps. Such translocation forms could have a significant contribution to locating gene control traits for yield, quality, agronomy, and resistance to abiotic and biotic stress factors. Finally, translocation lines of hexaploid triticale, lacking a 5BL chromosome segment carrying the *Ph1* gene locus (38T+D5BS-5BL.5RL), could be used for the induction of homeologous recombination, which is a crucial issue in chromosome engineering (Sears, 1981), considering the introduction of alien chromatin fragments with desirable genes or loci from related species directly into triticale.

## Author contributions

MK initiated the project and designed the study. MK, HW, and JB obtained the hybrid plants. AŚ and HP produced the doubled haploid lines. JM and MM made the mitotic chromosome preparations followed by FISH analysis. MK performed the GISH/FISH analysis, meiotic analysis and statistics. MK wrote the paper.

## Funding

This work was supported by the National Science Centre, Kraków, Poland (grant NCN SONATA 6; 2013/11/D/NZ9/02719).

### Conflict of interest statement

The authors declare that the research was conducted in the absence of any commercial or financial relationships that could be construed as a potential conflict of interest. The reviewer MM and handling Editor declared their shared affiliation, and the handling Editor states that the process nevertheless met the standards of a fair and objective review.
